# Validation of Point-of-Care Glucose Testing for Diagnosis of Type 2 Diabetes

**DOI:** 10.1155/2013/206309

**Published:** 2013-12-08

**Authors:** Marijana Vučić Lovrenčić, Vanja Radišić Biljak, Sandra Božičević, Edita Pape-Medvidović, Spomenka Ljubić

**Affiliations:** ^1^Department of Laboratory Medicine, Institute of Medical Biochemistry and Laboratory Medicine, Merkur University Hospital, 10000 Zagreb, Croatia; ^2^Vuk Vrhovac University Clinic, Merkur University Hospital, 10000 Zagreb, Croatia

## Abstract

Point-of-care (POC) glucose technology is currently considered to be insufficiently accurate for the diagnosis of diabetes. The objective of this study was to investigate the diagnostic accuracy of an innovative, interference-resistant POC glucose meter (StatStrip glucose hospital meter, Nova Biomedical, USA) in subjects with a previous history of dysglycaemia, undergoing a 75 g diagnostic oral glucose tolerance test (oGTT). Venous and capillary blood sampling for the reference laboratory procedure (RLP) and POC-glucose measurement was carried out at fasting and 2 h oGTT, and categories of glucose tolerance were classified according to 2006 WHO diagnostic criteria for the respective sample type. We found an excellent between-method correlation at fasting (*r* = 0.9681, *P* < 0.0001) and 2 h oGTT (*r* = 0.9768, *P* < 0.0001) and an almost perfect diagnostic agreement (weighted Kappa = 0.858). Within a total of 237 study subjects, 137 were diagnosed with diabetes with RLP, and only 6 of them were reclassified as having glucose intolerance with POC. The diagnostic performance of POC-fasting glucose in discriminating between the normal and any category of disturbed glucose tolerance did not differ from the RLP (*P* = 0.081). Results of this study indicate that StatStrip POC glucose meter could serve as a reliable tool for the diabetes diagnosis, particularly in primary healthcare facilities with dispersed blood sampling services.

## 1. Introduction

The presence of hyperglycaemia, as measured by standard laboratory procedures, is a pivotal biochemical marker used for the diagnosis of diabetes mellitus. Current diagnostic criteria imply a diagnosis of diabetes if any of the following glucose-based criteria are met: fasting venous plasma glucose (FPG) ≥7.0 mmol/L, 2 h venous plasma glucose (2 h PG) during a 75 g oral glucose tolerance test (oGTT) ≥11.1 mmol/L, or casual venous plasma glucose ≥11.1 mmol/L associated with clinical symptoms of hyperglycaemia [1, 2]. The utility of the recently recommended use of HbA_1c_ in terms of diabetes diagnosis, with a diagnostic threshold of ≥6.5% (48 mmol/mol), still remains to be validated [1].

Preanalytical sample handling is critical in obtaining accurate plasma glucose values, because of the need to reduce the influence of *in vitro* glycolysis. Any delay in sample processing may result in a reduction of plasma glucose values and missclassification of asymptomatic patients with diabetes and intermediate hyperglycaemia. Thus, a rigorous preanalytical procedure assuring glucose stability is recommended, involving either blood sampling with the addition of a glycolytic inhibitor which can only partly prevent *in vitro* glycolysis or immediate cooling of samples and centrifugation, followed by plasma separation within 30 minutes from the time of venipuncture [1–3]. As regards to analytical quality, it is recommended that venous plasma glucose for diagnosis of diabetes should be measured in an accredited laboratory, using an automated procedure with analytical imprecision <2.9%, a bias <2.0%, and a total error <6.9% [3]. While most of the laboratories achieve analytical claims, compliance to the recommended preanalytical procedure is far less easily accomplished [4] and variable preanalytical procedures have been identified as a serious drawback for the efficient diabetes diagnosis, particularly in primary healthcare facilities [5].

The use of point-of-care (POC) glucose meters in self-monitoring of blood glucose is an invaluable tool for glycaemic control management in patients with diabetes. However, despite many obvious advantages (low sample volume, short turnaround time, and availability outside of the laboratory) and ongoing improvements in analytical performance, there is limited evidence on the possible use of POC glucose testing for diagnostic purposes, and results collected so far remained controversial [6–8]. Although some evidence from epidemiological studies suggest that POC glucose meters could be helpful in screening for diabetes, particularly in remote areas [9–11], their use in the diagnosis of diabetes is not recommended, due to both insufficient precision and accuracy and the inherent, sample-dependent flaw of results [1–3]. Namely, whole blood glucose, as measured by POC glucose meters, is approximately 11% lower than plasma glucose, but the difference is hematocrit dependent. Furthermore, due to the arteriovenous difference in postprandial, but not fasting, state, specific, capillary-plasma-based thresholds must be used for diagnostic purposes [2]. While the recommended reporting of POC-glucose results to the plasma glucose equivalents enabled capillary-plasma-based diagnosis and improved comparability to the laboratory results [12], conversion of results by using mathematical algorithms may still be inaccurate in any clinical situation associated to either hemoconcentration or hemodilution, and appropriate correction for hematocrit-associated interference remains one of the most prominent challenges in POC glucose technology. A new generation of POC glucose meter, with unique hematocrit- and drug-interference-resistant technology (StatStrip glucose hospital meter, Nova Biomedical, USA), has been reported to be an accurate and reliable tool for glucose monitoring in a wide range of clinical settings [13–16].

The primary objective of our study was to investigate whether state-of-the-art POC glucose technology could be used as a diagnostic tool for type 2 diabetes mellitus and intermediate hyperglycaemia [2]. We hypothesized that an innovative POC-glucose technology might have achieved sufficient analytical accuracy for diagnostic purposes. To answer this question, we validated the diagnostic accuracy of the capillary plasma glucose measured with StatStrip glucose hospital meter against venous plasma glucose measured with reference laboratory procedure, in asymptomatic subjects with a previous history of dysglycaemia, undergoing 75 g oGTT according to 2006 World Health Organisation (WHO) recommendation [2].

## 2. Materials and Methods

### 2.1. Subjects

Adult subjects, referred to the Vuk Vrhovac University Clinic from April 2012 till April 2013 for the diagnosis of diabetes, were consecutively enrolled in this study. The subjects were either screened by their family physicians and found to have fasting hyperglycaemia (>6.1 mmol/L) or were diagnosed with intermediate hyperglycaemia (impaired fasting glucose (IFG) or impaired glucose tolerance (IGT)), as assessed by oGTT on their previous visits to our Clinic. Pregnant women, referred for the diagnosis of gestational diabetes, and subjects receiving any kind of medication affecting glucose metabolism (e.g., corticosteroids, oral hypoglycaemic agents) were not included in this study.

The study was designed in accordance with the principles of the Declaration of Helsinki and Good Clinical Practice and approved by the hospital's ethics committee. A written informed consent was obtained from all subjects.

### 2.2. Laboratory Methods

All laboratory procedures (preanalytical, analytical, and postanalytical) were performed by educated laboratory personnel according to standard operating procedures for the accredited laboratory (ISO 15189 Medical laboratories—particular requirements for quality and competence).

In the morning (7.00–9.00 a.m.) after an overnight fast, consenting subjects underwent standard procedure for 75 g oGTT [2]. Venous blood was sampled in heparinized tubes (Becton Dickinson, USA) at fasting and 2 hours after peroral ingestion of 75 g glucose dissolved in 250 mL plain water. To prevent the influence of *in vitro* glycolysis, heparinized blood samples were immediately refrigerated (2–8°C) and plasma separated from cells after centrifugation (3000 ×g, 10 min) not later than 30 minutes from venipuncture. Commercially available automated hexokinase assay (BC-AU400, Beckman-Coulter, USA), accredited according to ISO15189 standard, with a calibration traceable to the Standard Reference Material issued by the National Institute of Standards and Technology (NIST SRM 965), was used as the reference laboratory procedure for venous plasma glucose measurement (RLP). Within- and between-run imprecision, expressed as coefficient of variation (CV), was 0.95 and 1.18%, respectively.

Immediately after venipuncture, at each time point of oGTT, capillary blood was sampled by pricking fourth finger of nondominant hand, and point-of-care (POC) glucose was measured in duplicate, by using two StatStrip glucose meters and two different lots of reagent strips. StatStrip reagent strips are equipped with a modified glucose oxidase-based amperometric test system with unique hematocrit/chemical/drug-interference blanking system and sampling control. Results are expressed in plasma-glucose equivalents, according to current recommendations [1, 12]. As previously reported, within- and between-run imprecision was 2.0 and 2.4%, respectively [15].

Hematocrit was determined by an automated blood counter (Advia120, Siemens Diagnostic Solutions, USA), and HbA_1c_ was measured with a commercially available immunoturbidimetric procedure (TinaQuant, Cobas Integra-400Plus, Roche Diagnostics, Germany) traceable to the IFCC reference system, with results reported in both NGSP-conventional (%) and SI (mmol/mol) units. Fasting EDTA-blood samples were obtained for these analyses.

### 2.3. Classification of Glycaemia

Glycaemic status was classified according to the 2006 WHO diagnostic criteria for diabetes and intermediate hyperglycaemia. Based on FPG and 2 h PG, subjects were classified as either having normal glucose tolerance (NGT), impaired fasting glucose (IFG), impaired glucose tolerance (IGT), or diabetes mellitus (DM), by using sample type-related classification criteria for venous and capillary plasma, respectively [2].

### 2.4. Statistical Analysis

Data are presented as mean ± SD. Pearson's correlation and Passing-Bablok regression analysis were used for the analytical between-methods comparison. After testing for normality, differences between the categories of glycaemia were evaluated with ANOVA, followed by Student-Newman-Keuls test for pairwise comparisons, while the differences between POC- and RLP-glucose results were analysed with paired samples students's *t*-test. Possible influence of hematocrit on between-method bias was assessed with linear regression analysis. Any *P* value of <0.05 was considered significant.

Bland-Altman analysis was used to determine between-meter reproducibility, as well as bias and limits of agreement between POC- and RLP-glucose results. Concordance between the methods across the categories of glycaemia was assessed using *κ* (kappa) value obtained by interrater agreement analysis. The strength of agreement was interpreted as poor (*κ* < 0.20), fair (*κ* = 0.21–0.40), moderate (*κ* = 0.41–0.60), good (*κ* = 0.61–0.80), and very good (*κ* = 0.81–1.00). Diagnostic performances of the POC- and RLP-procedure to discriminate between normal and abnormal fasting glycaemia were compared with receiver operating characteristic (ROC) curve analysis. Power analysis (power = 80%, alpha = 0.05) has indicated that the minimal required sample size to detect significant differences between the methods at the upper level of normoglycaemia (6.1 mmol/L) and allowable bias (2%) is 38 subjects in each group (normoglycaemia versus any hyperglycaemia).

MedCalc statistical software, version 9.4.2.0 (MedCalc Software, Belgium), was used for the data analysis.

## 3. Results 

### 3.1. Analytical Results

Between-meter/reagent-strip *bias*, assessed by Bland-Altman analysis, was not significant: 0.1147 mmol/L (95% CI: −0.00671 to 0.2167) for the FPG and 0.1318 (95% CI: −0.0713 to 0.2432) for the 2 h PG values, respectively. Mean POC-plasma glucose from duplicate POC measurements was used for further statistical analyses. The correlation between RLP- and POC-FPG values was excellent (*r* = 0.9681, *P* < 0.0001), and Passing-Bablok regression analysis could not demonstrate significant difference between the methods (regression equation: *y* = −0.1000 + 1.0000*x*, 95% CI for intercept and slope: −0.3000 to 0.1806 and 0.9677 to 1.0323, resp.). With an excellent correlation maintained (*r* = 0.9768, *P* < 0.0001), 2 h PG values were found to be significantly different between the analytical methods (regression equation: *y* = −0.9477 + 1.0682*x*, 95% CI for intercept and slope: −1.2300 to −0.6117 and 1.0360 to 1.1000, resp.).

### 3.2. Clinical Results

Out of 241 patients consenting to participate, 3 dropped out because of discontinuation of oGTT due to nausea, and one patient developed clinically significant hypoglycaemia (2 h PG = 2.4 mmol/L) requiring medical intervention. Their results were excluded from the further analysis.

A total of 237 consenting subjects (41% males) with complete oGTT were included in this study. Glycaemic status was classified according to the FPG and 2 h PG values measured in venous plasma by the reference laboratory procedure (FPG-RLP and 2 hG-RLP, resp.), and results are summarized in Table 1. Patients with diabetes mellitus had significantly higher (*P* < 0.05) FPG and 2 h PG, as well as HbA_1c_, than subjects classified as having either NGT, IFG or IGT. The difference between the RLP- and POC-FPG was not significant in either subgroup of glycaemia (*P* = 0.3319, 0.1067, 0.3048, and 0.2825 for the NGT, IFG, IGT, and DM subgroups, resp.), while a weak but significant difference (*P* = 0.0326) was found in the entire study group. 2 h PG-POC-glucose was significantly higher than RLP-glucose in the entire study group (*P* < 0.0001), as well as subgroups with NGT and DM (*P* < 0.0001 and *P* < 0.05, resp.; Table 1). Hematocrit was significantly higher in DM group when compared to IGT and NGT but had no influence on the bias between the glucose values obtained by the two methods, neither at fasting (*P* = 0.457) nor at 2 h oGTT (*P* = 0.844).

**Figure 1:**
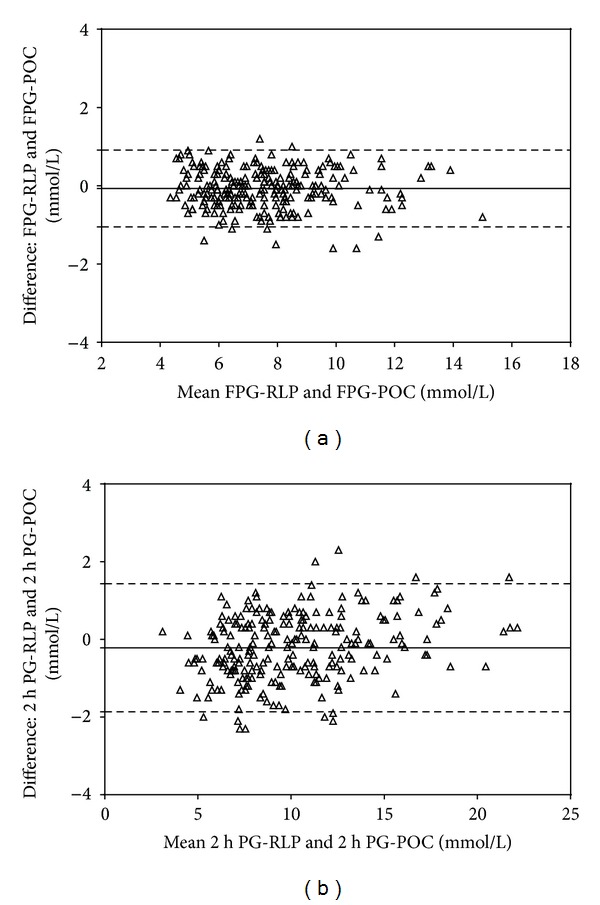
Bland-Altman plot of differences between (a) fasting plasma glucose (FPG) and (b) plasma glucose at 2 h oGTT (2 h PG), as measured with reference laboratory procedure (RLP) and point-of-care (POC) testing.

Bland-Altman analysis revealed a slight bias between the RLP- and POC-FPG with a mean difference of −0.06975 ± 0.5006 mmol/L (95% CI: −0.1337 to −0.005828 mmol/L; Figure 1(a)). Between-method mean difference at 2 h PG was −0.2219 ± 0.8382 mmol/L (95% CI: −0.3292 to −0.1147 mmol/L; Figure 1(b)).

### 3.3. Diagnostic Results

Study subjects were classified into categories of glycaemia using appropriate diagnostic thresholds for the venous and capillary plasma samples, according to the 2006 WHO criteria (Table 2). Interrater agreement analysis showed a very good agreement (weighted Kappa = 0.858) between the RLP- and POC-plasma glucose results when classifying subjects into categories of glycaemia. With capillary plasma-POC-based classification compared to the venous plasma-RLP as the reference classification, we found 96.5%, 65.5%, 60.9%, and 86% concordant cases for the categories of DM, IFG, IGT, and NGT, respectively. Discordant cases of DM and NGT were reclassified either between IGT or IFG category, whereas discordant cases of IFG and IGT were reclassified mostly between each other or DM category, while 5/27 (17%) cases of IFG and 3/28 (10.7%) cases of IGT were reclassified as NGT, based on capillary plasma-POC values (Table 2).

Finally, the ROC-curve analysis showed an excellent diagnostic performance of the POC-FPG, in discriminating between the NGT and any category of dysglycaemia, and pairwise comparison of ROC curves could not demonstrate the difference between the RLP-venous plasma and POC-capillary plasma AUCs (0.969, SE = 0.00992 and 0.952, and SE = 0.0129, resp.; *P* = 0.081).

## 4. Discussion

The analytical accuracy of POC-glucose meters has been a matter of extensive evaluation; however, due to the use of unharmonized evaluation protocols and clinical standards for acceptable performance, it is difficult to achieve an objective insight into reliability and quality of performance of POC-glucose meters within diverse clinical situations [17–19]. Until recently, rigorous analytical quality specifications issued for plasma glucose methods used for diabetes diagnosis were beyond the reach of POC-glucose technology. Previous reports indicated that StatStrip POC hospital glucose meter not only fulfills the claims for analytical quality, but serves as a reliable tool for accurate glucose measurement in the most demanding clinical settings, including intensive care unit [14–16, 20]. This performance data prompted us to choose StatStrip as the POC-glucose meter for this study. To our knowledge, this is the first study reporting on an excellent performance of the state-of-the-art POC-glucose technology, fully comparable to the accredited laboratory procedure, for the diagnosis of type 2 diabetes.

Despite its well-known disadvantages regarding impracticability and poor reproducibility, a 75 g oGTT still remains a standard diagnostic procedure for diagnosing diabetes and impaired glucose tolerance in patients with fasting plasma glucose ≤7.0 mmol/L [2]. In our study, 237 subjects with referring diagnosis of dysglycaemia underwent oGTT and 137 were diagnosed with type 2 diabetes, which is of no surprise considering that our clinic is the most prominent national healthcare facility specialized for diabetes care. We found an excellent correlation between the POC- and RLP-glucose values at each point of oGTT. A slight bias observed between the methods in FPG (Figure 1(a)) is consistent with previous reports in the general population [11], but this had no influence on the diagnostic performance of the POC-FPG in discriminating between normal glucose tolerance and any degree of dysglycaemia, as assessed with ROC-curve analysis. Between-method bias at 2 h oGTT was more pronounced (Figure 1(b)) reflecting well-known differences between the capillary and venous blood in postprandial conditions, which is recognized in the WHO diagnostic criteria for capillary plasma [2]. However, it is worth noticing that the most pronounced difference in 2 h PG was observed in the NGT group, maintained with less degree in the DM group, while no between-method differences could be demonstrated in the subgroups of intermediate hyperglycaemia (IFG and IGT). It could be argued that various degrees of peripheral insulin resistance and/or disturbed insulin action, both involved in type 2 diabetes pathophysiology, might be responsible for disturbed metabolic activity, which is reflected in the difference in distribution of glucose between the arterial and venous blood compartments in the postprandial state [20]. Regardless of the mechanisms involved, our results are consistent with previous findings [11, 20, 21], indicating that 2006 WHO diagnostic thresholds for the capillary plasma glucose, based on simple mathematical conversion by adding 1.2 and 1.1 mmol/L to the venous plasma thresholds for IGT and DM categories, could benefit from refinement according to the degree of glucose intolerance.

Not only analytical accuracy, but also between meter/reagent strip-reproducibility, as well as resistance to the well-known interferences, must be validated when considering the use of POC-glucose technology for the diabetes diagnosis. No significant differences could be demonstrated between the two StatStrip meters and two reagent strip lots in our study, and hematocrit had no influence on the bias between the RLP- and POC-PG results at either point of oGTT.

The main purpose of our study, however, was to assess the diagnostic accuracy of a modern day POC glucose technology for type 2 diabetes diagnosis. In our study population, less than 5% (6/137) of discordant cases were found in the DM subgroup with POC-capillary plasma glucose, and these were reclassified as either having IFG (2) or IGT (4). Also, none of 6 discordant cases (14%) in the group of NGT were reclassified as having DM or IGT. Relatively lower degree of concordance in the subgroups of intermediate hyperglycaemia (65.5% for IFG and 60.9% for IGT) could be explained, at least in part, by the variable magnitude of between-method difference observed in different categories of glycaemia (Table 1), which, as previously mentioned, are not addressed by the 2006 WHO-recommended classification criteria used in our study.

Previous study evidence could not demonstrate sufficient accuracy of POC glucose testing for use for diagnostic purposes [6, 7], and their performance was found to be inferior to laboratory glucose measurement for the epidemiological screening in indigenous population [8]. However, in a recent report, Ritchie et al. emphasized the utility of modern POC-glucose testing for screening purposes in a high-risk rural population [10], confirming previous evidence from epidemiological studies in remote areas [9], and the general population [11]. This data, as well as our evidence that shows that state-of-the-art POC-glucose technology can be used reliably as a diagnostic tool as well, demonstrates that the evolving technology of POC-glucose testing can offer many advantages beyond glucose monitoring. *In vitro* glycolysis, as the most prominent source of variability affecting plasma glucose values and classification of glycaemic status, is completely avoided by the use of POC technology. This is particularly important in primary healthcare facilities with dispersed blood sampling services, often lacking equipment necessary for the appropriate sample handling and preparation. Moreover, immediate availability of results substantially improves the efficiency of the overall healthcare process associated with diabetes diagnosis, especially when timely procedure of oGTT is involved.

Our study provides sufficient evidence for further research on the validity of POC glucose technology for diagnosis of type 2 diabetes, with a multicentre evaluation at the level of primary healthcare as the most interesting task in the near future. Apart from diagnostic accuracy, the issue of cost-effectiveness should also be investigated before a general implementation of POC-glucose technology for the diagnosis of type 2 diabetes mellitus could be recommended.

## 5. Conclusions

Our study provides the first evidence on the comparable diagnostic performance of the StatStrip POC hospital glucose meter to the accredited laboratory glucose measurement procedure. Results of this study indicate that POC-glucose testing, provided that validated state-of-the-art technology fulfilling rigorous quality claims is used, could serve as an accurate and reliable tool for the diagnosis of type 2 diabetes.

## Figures and Tables

**Table 1:** Subject characteristics according to the 2006 WHO classification of glycaemia [2].

Variables	Subject category				
	DM	IFG	IGT	NGT	Total
*N*	137	29	28	43	237
M/F	62/75	12/17	11/17	14/29	99/138
Age (years)	59 ± 11	52 ± 15	55 ± 16	42 ± 15	55 ± 14
BMI (kg/m^2^)	30.7 ± 4.2	29.4 ± 4.0	29.9 ± 4.1	28.2 ± 4.2	29.3 ± 4.1
FPG-RLP (mmol/L)	8.7 ± 1.7^a,b,c^	6.4 ± 0.3^c,d^	6.2 ± 0.7^c,d^	5.3 ± 0.5^b,c,d^	7.5 ± 2.0
FPG-POC (mmol/L)	8.8 ± 1.7^a,b,c^	6.5 ± 0,5^c,d^	6.2 ± 0.8^b,d^	5.4 ± 0.6^b,c,d^	7.6 ± 2.0^†^
2 h PG-RLP (mmol/L)	11.9 ± 3.8^a,b,c^	6.7 ± 1.2^c,d^	9.8 ± 1.2^a,b,d^	6.3 ± 1.3^c,d^	9.9 ± 3.8
2 h PG-POC (mmol/L)	12.0 ± 3.5^a,b,c†^	6.9 ± 1.0^c,d^	9.9 ± 1.2^a,b,d^	6.8 ± 1.2^c,d††^	10.2 ± 3.6^††^
HbA_1c_ (%)	6.8 ± 1.1^a,b,c^	5.7 ± 0.35^d^	5.9 ± 0.72^d^	5.5 ± 0.32^d^	6.4 ± 1.07
HbA_1c_ (mmol/mol)	52 ± 12^a,b,c^	39 ± 4^d^	42 ± 8^d^	36 ± 7^d^	47 ± 12
Hct (L/L)	0.427 ± 0.034^a,c^	0.413 ± 0.027^d^	0.402 ± 0.031	0.40 ± 0.034^d^	0.419 ± 0.035

*P* < 0.05 versus ^a^NGT, ^b^IFG, ^c^IGT, and ^d^DM.

^†^ *P* < 0.05; ^††^ *P* < 0.0001 versus RLP.

**Table 2:** Classification of glycaemia based on plasma glucose results measured with reference laboratory procedure (RLP) and point-of-care (POC) glucose testing, according to the 2006 WHO criteria for venous and capillary plasma [2].

Capillary plasma-POC	Venous plasma-RLP				Capillary plasma-POC (*n*)
	DM	IFG	IGT	NGT	
DM	131	4	6	0	**141**
IFG	2	19	2	6	**29**
IGT	4	1	17	0	**22**
NGT	0	5	3	37	**45**
Venous plasma-RLP (*n*)	**137**	**29**	**28**	**43**	**237**
